# Virtual Coaches: Background, Theories, and Future Research Directions

**DOI:** 10.1007/s12599-022-00757-9

**Published:** 2022-07-13

**Authors:** Thure Georg Weimann, Hannes Schlieter, Alfred Benedikt Brendel

**Affiliations:** 1 Research Group Digital Health, TU Dresden, Dresden, Germany; 2 Chair of Business Informatics, esp. Intelligent Systems and Services, TU Dresden, Dresden, Germany

**Keywords:** Virtual coaches, Persuasive systems, Behavior change, Virtual assistants, Conversational agents, Foundational definition

## Introduction

Digitalization crosses all areas of life (Hess et al. 2014). Recent progress in artificial intelligence (AI) opens new potentials for further developments and improvements, with virtual coaching being a prime example. Virtual coaches (VCs) aim to optimize the user’s life by transforming cognition, affection, and behavior towards a stated goal. Since they emerged from the health and sports domain, a typical example are VCs in the form of digital avatars, which instruct physical exercises, shape health-related knowledge and provide motivational support to achieve the user’s goals (e.g., weight loss) (Ding et al. 2010; Tropea et al. 2019). Nonetheless, the application areas of VCs are versatile and exploring the potential areas (e.g., healthcare, work, finance, leisure, and environment) constitutes an essential topic of future research and development. According to Gartner’s hype cycle for human capital management technology, VCs are still in their infancy but are considered innovation triggers for the following years (Gartner, Inc. 2021). Specifically, VCs can be a replacement or complement for traditional human-to-human coaching scenarios and promise broad access to personalized coaching services independent of place and time (Graßmann and Schermuly 2021). As a result, VCs may contribute to solving challenges posed by an aging society and skilled labor shortage (European Commission 2016; Edwards and Cheok 2018). Last but not least, the recent COVID-19 pandemic additionally showcased the need for VCs as an alternative to traditional face-to-face interventions. Against this background and driven by the potential and promises of VCs, research has recently engaged in developing and understanding VC applications (Tropea et al. 2019; Lete et al. 2020; Graßmann and Schermuly 2021).

To introduce the concept in information systems (IS) research and provide a basis for researchers and practitioners alike, this catchword aims at providing a holistic view on VCs. The structure of this paper is as follows. Section 2 elaborates a definition, delimits VCs from related system classes, and proposes a research framework. Section 3 aggregates existing research into the framework and concludes with an outlook on future IS research perspectives.

## Conceptual Foundations

### Definition

Caused by the recency of the emergence, there is no unified definition of a VC and terms like “e-coach”, “AI coach” or “digital coach” are used synonymously in the literature (e.g., Tropea et al. 2019; Kamali et al. 2020; Graßmann and Schermuly 2021). Coaching (often used synonymously to the term “training”), in general, refers to the measures that help to transform someone from one state to another (Starr 2008, p. 4; Passmore and Lai 2020). It defines as “a conversation, or series of conversations, that one person has with another”*,* where a coaching conversation is considered to be effective when it “influences someone’s understanding, learning, behaviour and progress” (Starr 2016, p. 7). Thus, coaching has its roots in social psychology that studies how the interlinked concepts of cognition, affection, and behavior can be transformed through the influence of other humans in a social context (Allport 1968). Similar to nudging, coaching can be justified by improvements for the individual (pro-self) or the society in general (pro-social) (Lembcke et al. 2019). Even though there are similarities, Kamphorst (2017) argues that users should be at least aware of the coaching, which is often not the case with nudging. While different behaviors are associated with a coach, for instance, knowledge transmission or feedback provision that overlap with other developmental relationships (e.g., tutoring or mentoring), coaching emphasizes building a trustworthy relationship to the coachee and a continuous goal setting (D’Abate et al. 2003; Passmore and Lai 2020). Therefore, coaching is considered more outcome or performance-oriented than mentoring or tutoring. It can be understood as a cycle where the performance of the individual is evaluated to suggest actions that have worked and reduce or eliminate actions that were not successful in the subsequent cycle (Grant 2012). Consequently, the aspect of longevity is essential, meaning that multiple interactions with the coach are required to achieve and maintain a transformation (Passmore and Lai 2020).

Driven by new technological possibilities, some authors picked up the idea of digitalizing the human coach and suggested corresponding definitions. There is a broad understanding of virtual coaching in the literature that includes any form of coaching using electronic media. For example, Geissler et al. (2014) characterize virtual coaching as “coaching mediated through modern media […] by replacing face to face communication with modern media”. Consequently, this understanding includes software that functions as a synchronous or asynchronous communication medium to contact a human coach (e.g., video telephony or e-mail) and autonomous software systems that conduct coaching themselves. The latter one refers to a narrow understanding of VCs as software agents, i.e., autonomous systems, that provide coaching functionality (Kamphorst 2017; Scholten et al. 2017). A fundamental characteristic of a VC is “context awareness” that enables the coach to understand the user’s situation, define appropriate goals and actions, monitor progress, and act proactively (Ding et al. 2010). Thus, VCs go beyond traditional (non-intelligent) e-learning software that presents static content on a pre-determined curriculum to the user by adapting to the context and encouraging behavior changes. Digital ubiquity raises context awareness of the coach to a new level and renders it possible to gather data via sensors or direct user inputs throughout the user’s life.

**Fig. 1:**
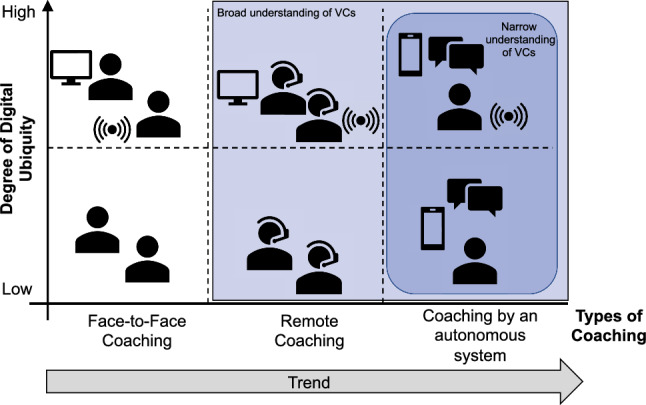
Classification of coaching types

To summarize, three types of coaching can be distinguished (see Fig. 1): face-to-face coaching, remote coaching, and coaching by autonomous systems. While coaching as a face-to-face conversation is the traditional and arguably most common format, virtual coaching refers to remote coaching in a broader sense and coaching conducted by autonomous systems in a narrow sense. Predominantly driven by progress in the field of AI, there is a clear trend towards the last type of coaching (Tropea et al. 2019; Lete et al. 2020; Graßmann and Schermuly 2021). Nevertheless, combining face-to-face coaching with remote or autonomous coaching in an alternating way is still conceivable and referred to by Geissler et al. (2014) as “Blended coaching”. The different types can be enriched further with data gathered by digital devices which are placed on the user’s body (also called wearables), in the user’s environment (e.g., smart objects), or sensed data stored in databases (e.g., weather data) (Lete et al. 2020). We call this data enrichment of the coaching process that enables a high degree of context-awareness “digital ubiquity”.

For this catchword, we follow a rather broad understanding but distinguish VCs from software that *solely* provides communication mechanisms for connecting the human coach and coachee by being at least partially autonomous. Thus, the degree of autonomy may vary on a continuum, creating different opportunities for collaborating with the human coach (see Sect. 3 (D)). Independent of the VC’s degree of autonomy, the human coach remains the configuring instance before deployment and origin of the system’s intelligence. We define VCs as *partially to fully autonomous software systems that target a transformation of the user’s cognition, affection, and behavior over multiple interactions, justified by improvements for the user or society towards a particular goal, with a continuous adaptation of the coaching actions depending on the context.*

### Related System Classes and Differences

**Fig. 2:**
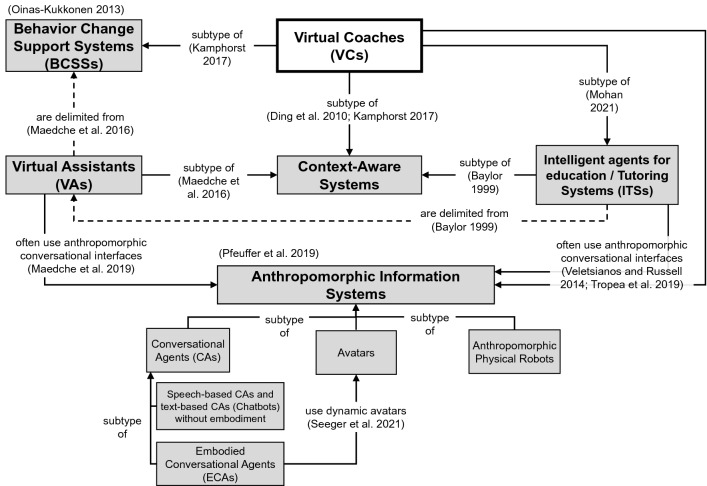
Relationship of virtual coaches to other system classes

A related concept of VCs are virtual assistants (VAs) that are also referred to as “AI-based digital assistants”, “advanced user assistance systems”, or “personal digital assistants” in the literature (Maedche et al. 2016, 2019; Sarikaya 2017). Widespread instances of this system class are speech-based assistants like “Apple Siri” or “Amazon Alexa” (Diederich et al. 2019). Both VCs and VAs frequently use anthropomorphic conversational interfaces and are considered context-aware systems. However, significant differences lead to unique challenges when designing VCs (see Fig. 2 and Table 1).

**Table 1:**
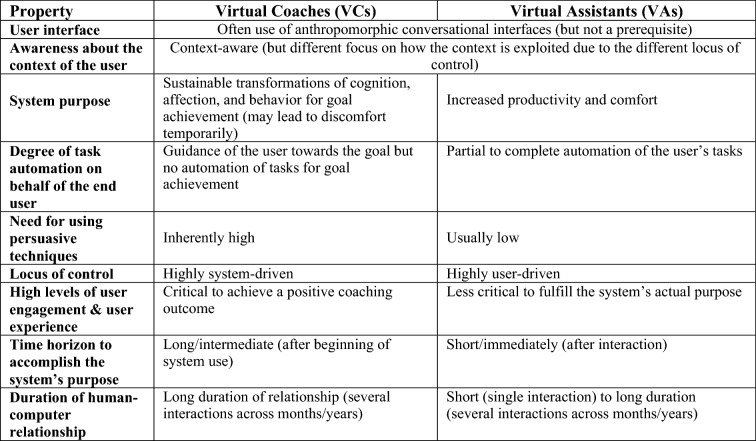
Shared properties and differences between Virtual Coaches and Assistants

VAs are systems that aim to facilitate routine tasks through partial to full automation in order to increase productivity and comfort of the users so that they can devote more time to other tasks (Sarikaya 2017; Maedche et al. 2019; Budzinski et al. 2019). Therefore, they have also been associated with the metaphor of a “butler” in the literature (Pfeuffer et al. 2019; Budzinski et al. 2019), but even in the early days of intelligent systems Baylor (1999) endorsed a differentiation when talking about a “coach” or “tutor”. Generally speaking, VCs do not aim to automate tasks for the user (coachee). Instead, they aim to automate tasks on behalf of the human coach. As mentioned, VCs are intended to be transformative, which can lead to discomfort and additional users’ efforts. For example, increasing physical activity, quitting smoking, and following healthier nutrition can make users feel uncomfortable breaking old habits. Therefore, VCs are a subtype of so-called “Behavior change support systems” (BCSSs) and “Intelligent Tutoring Systems” (ITSs) (Kamphorst 2017; Mohan 2021). BCSSs distinguish from other types of IS according to Oinas-Kukkonen (2013), in that they deliberately target cognitive, affective, and behavioral transformations by using persuasive techniques without deceiving or coercing the user to adopt a certain behavior. ITSs, on the other hand, aim to convey knowledge to the user and form cognitive skills (Baylor 1999; Mohan 2021). Considering that knowledge transfer impacts behavior change and is an integral part of coaching (but not all of it), designing VCs requires a holistic and interdisciplinary approach considering both currently rather disjointed research branches (Oinas-Kukkonen 2013; Mohan 2021). As a special type of BCSSs, VCs add the notion of social abilities and context awareness (Kamphorst 2017), the latter enabling the system to be proactive and cyclically re-adapt (i.e., self-learning). In contrast to BCSSs and VCs, VAs are generally delimited from persuasive systems, as Maedche et al. (2016) stated. However, a special case can be VAs that assist users in finding the right product by minimizing search costs and that might also use persuasive techniques to sell target products (Yu et al. 2011). Arguably, when buying a certain product once, there is no learning and gradual progress over multiple interactions, which delimits such systems from VCs. Nonetheless, one could think about a VC for achieving an eco-friendly lifestyle by purposefully buying sustainable products.

According to Følstad et al. (2019), another difference between VCs and VAs refers to the leader role of the dialogue (“locus of control”). While current VAs are highly user-driven and, for example, help to look up information if needed by the user or control devices as a reaction to the user’s command, VCs are mainly driven by the system and guide the user through a personalized and sequential coaching program (Følstad et al. 2019). Therefore, the VC needs to be proactive by anticipating opportune moments to interact with the user based on the observed context. For example, while working or driving a car, the user is usually not receptive to messages from the VC and system interaction might even pose a danger (op den Akker et al. 2015; Künzler et al. 2019). Another unique characteristic of VCs is a strong focus on increasing and maintaining high levels of user engagement and user experience over the long term, as continuous use of the system is critical for a positive coaching outcome (Bickmore and Picard 2005; Oinas-Kukkonen 2013). The effect of the VC only becomes apparent after a more extended or intermediate period of time, while the time horizon to accomplish the VAs actual system purpose is shortly or even immediately after interaction (e.g., ask for the weather). Therefore, VCs are always intended as systems for long-term use over several months or years as transformations take time. VAs, in contrast, may also be intended for long-term use, but there are use cases such as product search where the human–computer relationship can be short-lived (Yu et al. 2011).

### Research Framework

**Fig. 3:**
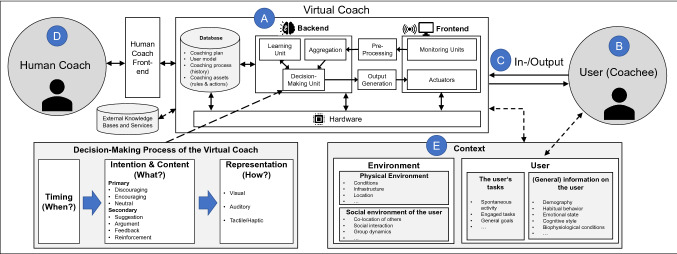
Research framework and building blocks of virtual coaches

Building on this definition, we develop a research framework that integrates and harmonizes the conceptual views on VCs outlined in prior literature (Schmidt et al. 1999; op den Akker et al. 2015; Sarikaya 2017; Ochoa and Gutierrez 2018; Maedche et al. 2019; Diederich et al. 2022). In our framework (see Fig. 3), we identify five central aspects of common VC scenarios. The core of every VC scenario is the application system **(A)** containing interfaces, data storage, and intelligence to process data, to trigger and monitor coaching activities. This system is embedded in a context **(E)** and interacts **(C)** with the user **(B)**. The VC is initialized by a human coach **(D)** or an existing knowledge base. Each aspect will be explained in detail and discussed against the background of prior research and opportunities for future research (Sect. 3).

In general, VCs can be structured as frontend-, backend- and underlying hardware components, where each of these components represents its own research area **(A)**. The front-end provides in- and/or output mechanisms and can be distributed across several hardware components to obtain multimodal interaction. Monitoring units capture the contextual data inputs, which are then pre-processed and forwarded to the backend. The actuators are the counterpart to the monitoring units and conduct the generated coaching actions (e.g., send a notification message). Pre-processing inputs and generating outputs may have a mediating role and can be conceptually assigned to the backend or frontend. Although the communication with the VC is always bidirectional due to the cyclic nature of the coaching process, there might exist hardware components that solely output information (e.g., vibration wristband as tactile feedback) or process inputs (e.g., heart rate sensor). The backend of the VC represents the actual intelligence and database of the coach. It decides about appropriate coaching actions based on the aggregated contextual data and historical data. A learning unit may adapt the coaching plan, the user model, and the rules by itself (i.e., self-learning) as more knowledge on the user and user groups are gained in each cycle (Ochoa and Gutierrez 2018). As proposed by op den Akker et al. (2015), the decision-making process of the coach can be structured by determining the timing of the coaching message (when?), the actual intention and content (what?), and the representation format of the message (how?). For example, the user can interact with a mobile phone app that provides real-time feedback messages during physical activity based on data received by an external heart rate sensor. When a user reduces effort during a workout, the VC has to decide to either motivate the user to increase activity (encouraging), to slow down the workout because the user might be on the brink of overexertion (discouraging), or to send a neutral message. Afterward, the VC needs to decide on a secondary intention (suggestion, argument, feedback, or reinforcement) before determining the actual content of the message. In the last step, the VC selects an appropriate representation format of the message (visual, auditory, and tactile/haptic feedback). Each step of the decision-making process can represent a separate research topic (e.g., investigating algorithms for determining suitable timings).

Regarding the user, we distinguish between research that focusses on a psychological understanding of the user (e.g., how can changes in affection, cognition, and behavior be explained?) **(B)** and research that focuses on the interaction design (e.g., what are the effects of certain VC outputs?) based on psychological and technical considerations **(C)**. Note that these research approaches are not disjointed but constitute different approaches and perspectives on VCs, and research results can be interrelated (Baskerville et al. 2018). Typically, the coaching program is pre-defined by a domain expert (the human coach) (Gand et al. 2021). Thus, research on the interplay of a human coach and the VC is focused on integrating explicit and implicit expert knowledge into the system **(D)**. Lastly, the whole socio-technical system is embedded into a particular context (e.g., healthcare, finance, etc.) with corresponding variables determining how the coach should react in specific situations and adapt the coaching schemes for the coachee. The context can be broadly conceptualized as factors related to the user (general information on the user, information regarding the user’s tasks) and factors related to the environment (physical and social) (Schmidt et al. 1999).

**Fig. 4:**
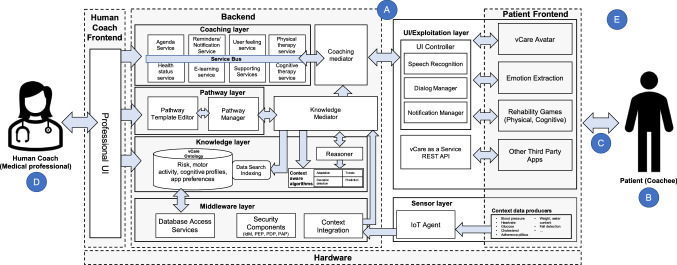
Exemplary architecture of a virtual coach for rehabilitation of older adults (adapted from Kyriazakos et al. (2020))

To provide an example application of the framework for a real-world VC, Fig. 4 illustrates the architecture of a VC for rehabilitation of older adults by Kyriazakos et al. (2020) and shows how the different building blocks of VCs (Fig. 3) may be designed and interact in practice. The scope of the VC (A) is to process care pathways for rehabilitation and provide personalized coaching recommendations. Therefore, they implemented a multi-layered structure consisting of a coaching layer, pathway layer, knowledge layer, and a middleware layer in the backend next to a user interface layer in the frontend. The user (B) interacts with a humanoid avatar representing the VC (C). Additionally, there is a dedicated user interface for medical professionals (i.e., the human coaches) (D) where the coaching pathways, knowledge base, and different services can be monitored and tailored for the user. Multiple internet of things (IoT) devices (e.g., blood pressure monitor, heart rate sensor, or medication adherence pillbox) are used as context producers (E).

## Related Work and Opportunities for Future Research

In the following section, we present the current state of research and opportunities for future research based on the components of the research framework. Concerning the latter, research questions (RQ) are referenced and summarized in Table 3.

### (A) Virtual Coaching System–Frontend

Several different interface modalities may be chosen for interaction between user and VC, for example, graphical, auditory, tactile/haptic, or sensor-based user interfaces (Tropea et al. 2019). In general, the choice of interface depends on the task of the VC, and also, individuals with disabilities have to be considered. However, given the conversational nature of coaching (Starr 2016, p. 7), previous studies have found conversational agent (CA) interfaces that emulate interpersonal communication useful for VCs (Tropea et al. 2019). Here, the human coach is considered as inspiration for a human-like (also called anthropomorphic) software design. Even if a human-like design of the VC is not a prerequisite, current research builds extensively on knowledge from the field of anthropomorphic IS (Pfeuffer et al. 2019; Kang and Wei 2020).

While CAs as part of the popular VAs “Siri” or “Alexa” are purely speech-based, Embodied Conversational Agents (ECAs) are designed with a digital avatar as a visual representation (Diederich et al. 2019; Seeger et al. 2021). Notably, the agent’s embodiment allows interacting verbally and non-verbally (Cassell 2000). Due to their suitability for pedagogical tasks, they are also referred to as “pedagogical agents” in the literature and have been used as interfaces of intelligent tutoring systems (Warner 2012; Veletsianos and Russell 2014).

To facilitate the implementation of text- or speech-based CAs, a manifold of platforms emerged in recent years (e.g., Dialogflow or Azure Bot Service) that are often used to set up VAs but can also be used in the development of VCs (Diederich et al. 2019). With ECAs, which are due to the interplay of visual and auditory interface design even more complex to implement, there is comparatively less guidance. However, there are avatar model “construction kits”, animation libraries, and lip-sync plugins available that can be integrated into tools commonly used in game development (e.g., “Unity”) to avoid starting from scratch (see Llorach et al. 2019). The avatar can then be built as web-, desktop-, mobile- or even as an application for virtual/augmented reality (VR/AR) glasses and controlled in real-time. In particular, the deployment of VCs on multiple platforms will be increasingly relevant in the future as they allow combined coaching scenarios (RQ1). A promising but technically complex approach might also be to consider more than one coach (“multi coaches”) (Beinema et al. 2021). In this case, it has to be technically ensured that the coaches collaborate and do not contradict (RQ2).

### (A) Virtual Coaching System–Backend

The VC backend tasks can be described as processing inputs from the front-end, decision-making, data logging, and generating suitable outputs that are propagated back to the front-end (Sarikaya 2017; Ochoa and Gutierrez 2018; Kyriazakos et al. 2020). After the input data has been pre-processed and aggregated, a decision can be made. The current context, stored past experiences with the user and potential coaching actions to reach a specific goal (coaching plan) are considered to select a suitable action. Suppose the user interface takes the form of a CA. In that case, the decision-making is enriched by a dialog manager that determines the following dialog action and keeps track of the dialog flow to have a meaningful conversation (Griol et al. 2020).

While decision-making has been often implemented as static rules (e.g., if the activity level of the user is low, then send “go for a 30-min walk!”) that are triggered at fixed points in time and are limited in terms of personalization, current research focusses on learning abilities of the coach (Gonul et al. 2019). Machine learning (ML) methods make the VC more dynamic and adaptable to the user context (Philipp et al. 2019). In general, ML algorithms can be studied for all stages of the decision-making process (see Fig. 3). Coaching decisions could then be based on learned user preferences and interventions that have been successful in the past (e.g., activity recommendations), while the system is still able to detect changes in preferences to avoid habituation or intervention fatigue at the cost of user engagement (Gonul et al. 2019). ML methods can also be used to predict favorable timings of coaching messages that are associated with positive effects (also referred to as “states of receptivity”) (Künzler et al. 2019). Future studies could investigate advanced algorithms as part of the backend that may be able to infer novel and personalized coaching strategies. Particularly reinforcement learning, a subfield of ML that investigates self-learning algorithms (e.g., Multi-Armed Bandits or Q-Learning) that continuously learn by interacting with the environment, represents a fruitful area of research (RQ3) (Gonul et al. 2019; Philipp et al. 2019). From the same perspective, methods from the field of “Explainable Artificial Intelligence” (xAI) seem promising to increase the interpretability of “black box” coaching decisions and enhance user trust (RQ4) (Wanner et al. 2020). Also, data protection issues arise, esp. through the integration of several IoT devices. These aspects need to be addressed so that users have a positive attitude towards the system from the beginning. Likewise, safety aspects must be considered (RQ5) when the VC is used for tasks that may affect the user’s health condition (e.g., rehabilitation). To accelerate VCs’ implementation, Filler et al. (2015) developed an open-source platform called “MobileCoach”^1^ that can be extended or revised by application developers and serve as a starting point to build proprietary systems. In addition, the platform could motivate the study of generic VCs for facilitating other application scenarios by “simply” replacing the coaching plans and could give rise to new platform business models in the future (RQ6).

### (B) User (Coachee)

Considering that understanding, learning, and behavior change are the main intentions of coaching (Starr 2016, p. 7), the importance of psychological theories that explain these processes becomes obvious. An essential foundation is the “computers are social actors” (CASA) paradigm that has been established for more than twenty years now and grounds the idea that technology can influence cognition, affection, and behavior in the same way as humans do (Nass and Moon 2000). The CASA paradigm is supported by several empirical studies that indicate that humans apply the same social rules to computers as to humans. Besides the CASA paradigm, Cognitive Load Theory (Sweller 1994), which attempts to explain how learning can be facilitated through reducing cognitive overload, and Social Cognitive Theories (e.g., Bandura 1986), which consider learning as a social process, are particularly relevant in the literature on ITSs (Veletsianos and Russell 2014). In terms of VCs that target a health behavior change, there is even consensus in the literature that a foundation in behavioral theory can improve the success of the intervention but is often a missing ingredient in practice (Webb et al. 2010; Klonoff 2019). Theories of behavior and behavior change that are frequently referred to are i) the Social Cognitive Theory (Bandura 1986), ii) the Transtheoretical Model (Prochaska and Velicer 1997), iii) the Theory of Planned Behavior (Ajzen 1991), and iv) Self Determination Theory (Ryan and Deci 2000). However, these theories have different perspectives on behavior and behavior change. Social Cognitive Theory, for instance, assumes that there is a dynamic interaction between personal factors (esp. cognition and affect), behavior, and the environment. The theory emphasizes learning by observing a model in the environment (e.g., the piano coach demonstrates how to play the piano). In contrast, the transtheoretical model attempts to explain that behavior change consists of several stages with cognitive-affective and behavior-oriented processes. A comparatively new approach is the COM-B model by Michie et al. (2011), which describes a bidirectional relationship between the factors capability, opportunity, and motivation that influence behavior. To stimulate behavior, Michie et al. (2011) proposed several intervention functions that are linked to capabilities, motivation, and opportunities. For example, the coach could use persuasion, incentivization, or coercion to target the intervention point “motivation”. Therefore, intervention points and appropriate coaching actions must be technologically mapped on the VC side (A).

When designing a VC, there are several ways to use the implications of the theories mentioned above to justify design decisions. One approach is to derive design features from the behavior influencing variables presented in those theories. For example, Androutsou et al. (2020) use the COM-B model and associate educational material with the development of capabilities, notifications with opportunities, and badges/achievements for the user with motivation. The Social Cognitive Theory, for instance, motivates the inclusion of human “peer coaches” as similar models to the coachee for promoting social comparison (Colón-Semenza et al. 2018). Considering the lack of evidence and understanding about the effectiveness of social comparison features (e.g., sharing daily activity or direct messaging functions) (Arigo and Suls 2018), the integration of peer coaching elements in VCs is an area to be explored in future studies. Another possibility to involve theories is to derive variables and use them to “tailor” coaching content to the user’s situation. For example, based on the transtheoretical model, it might be beneficial to emphasize pro arguments of a target behavior (e.g., “physical activity will improve your health condition”), especially for users in the early stages of change (Prochaska and Velicer 1997).

In recent years, research regarding the understanding of forming habits, i.e., automatic behaviors (e.g., “go for a walk”) as a response to a particular context (“after getting up”), received considerable attention in health psychology (Lally and Gardner 2013). Given a lack of research on how systems should be designed to effectively support the habit formation process (RQ8) (Karppinen et al. 2018), future studies are needed to better understand how they could support these mechanisms. Table 2 summarizes the theories introduced in this and the following subsection and provides exemplary research questions for future studies.

### (C) User–Coach Interaction

Building a trustworthy and engaging long-term human–coach relationship is crucial if cognitive, affective and behavioral changes are attempted (Bickmore and Picard 2005). Of particular importance for VCs are the concepts of “working alliance” that is considered as the mutual trust to achieve a certain goal and origins from psychotherapy, as well as rapport (Scholten et al. 2017). Research indicates that for use cases in which rapport and trust between user and system are essential (like virtual coaching), ECAs tend to be preferred over disembodied agents (Scholten et al. 2017; Loveys et al. 2020). Because of their higher media richness, allowing verbal and non-verbal social cues to be conveyed (Schuetzler et al. 2018), they can evoke a greater “sense of human contact embodied in a medium” (Gefen and Straub 1997) that is called social presence. While a higher media richness of the VC might be beneficial, it can also pose a hurdle. For example, a mismatch of a realistic voice and a rather unrealistic avatar representation may negatively affect user acceptance (Mitchell et al. 2011). This effect can be explained by the uncanny valley theory, as discussed by Mori et al. (2012). Based on the CASA paradigm, anthropomorphic software design elements (also called social cues), such as giving the VC a name or a particular design of the visual appearance of the avatar, can trigger social reactions in humans (e.g., trust or liking) (Feine et al. 2019). A persuasive system design is of pivotal importance for the effectiveness of the VC, with the human–coach relationship being a central factor (Bickmore and Picard 2005; Ding et al. 2010; Kamphorst 2017). Research aspects of social cues are diverse and may comprise investigation of effects when using different degrees of realism, as cartoonized vs. more realistic coaches, different roles (e.g., peer and expert), or various communication styles of the coach (e.g., fact based vs. more explanations by the coach) (ter Stal et al. 2020). Given the longevity of the user–coach interaction, future studies should investigate the long-term effects of certain social cues (RQ9). For instance, it could be conceivable that some social cues lose their effect after a very short period of time, whereas others do not. The general challenge is to keep the user engaged in using the system and preserving the persuasiveness of the VC for building a long-term relationship (RQ10 & RQ11). The VC cannot have further impact if a user becomes bored and stops usage. Promising strategies to address both the system’s persuasiveness and user engagement are the integration of gaming elements such as badges and experience points (“gamification”) or entire games (“serious games”) (Deterding et al. 2011; Klock et al. 2020). In particular, the Persuasive System Design (PSD) Model by Oinas-Kukkonen and Harjumaa (2009) provides valuable implications for designing VCs by stating various design principles with concrete application examples. Michie et al. (2013) proposed a taxonomy of behavior change techniques (e.g., self-monitoring, feedback, or rewards) due to the origin in health psychology less technology-focused than the PSD model but which can inform the VC design as well. In recent years, other complementing frameworks have also emerged, for example, the Just-in-Time Adaptive Interventions (JITAIs) framework (Nahum-Shani et al. 2018). However, with regard to just-in-time interventions and an omnipresence of the coach, it is noteworthy to also think of burdens for the user in the sense of technostress (Rieder et al. 2020) that could negatively affect the coaching outcome. Future studies could compare in experimental settings a rudimentary system design with a digital ubiquitous VC regarding the coaching outcome and perceived concerns of the users (RQ12). Last but not least, it should be noted that when a system is able to influence cognition, affection, and behavior, potential ethical issues may arise. For example, the user could be systematically influenced to buy “extensions” for the VC or is otherwise not well coached. Future studies should be aware of these ethical concerns, critically reflect the design artifacts against this background and propose design approaches to address ethical aspects (RQ13).

### (D) Human Coach

A human coach and VC may collaborate in the sense of a hybrid intelligence (Dellermann et al. 2019). Possible collaboration modes between the human coach and VC may be classified as “assisted decision- making”, “verified decision-making” and “delegated decision-making” (Maedche et al. 2019). Assisted decision-making could be, for example, that the human coach is notified by the system in safety-critical situations to contact the coachee or adapt the system based on expert knowledge during runtime (e.g., define and adjust coaching plans or decision rules). Dedicated software tools referred to as “expert panels” have been developed that are able to control the VC (Androutsou et al. 2020). Similar to the user perspective, the human coach interface should be considered a key factor for the VC success and is therefore also an object of research (RQ14). For instance, incorrect coaching rules caused by an unintuitive and error-prone interface can jeopardize the application of an otherwise flawless VC. Against this background, adjacent research areas, such as process modeling (Gand et al. 2021), can provide a rich ground to understand the needs of and design for the human coach. Prospective research could elaborate approaches and methodologies to integrate expert knowledge into the VC easily (RQ15). However, making implicit knowledge explicit can be considered a major challenge (Gand et al. 2021). Furthermore, even when the process of converting implicit knowledge into explicit knowledge has been figured out, some human coaches might fear that the VC replace them entirely. Similar challenges can be observed in the change management literature (Bérubé et al. 2021) and can provide a potential starting ground for understanding the needs of human coaches.

### (E) Context

Awareness about the context of the user and the environment forms the foundation for suitable coaching actions and personalized adaptation of the system (Ding et al. 2010; Kamphorst 2017). There is a vast and growing body of research in the field of context-aware systems that can be structured along the areas of context acquisition, context modelling, context reasoning, and context dissemination (see Perera et al. 2013). For acquiring the context, the VC could use active sensing by asking the user (e.g., via buttons, input forms, or multiple choice options) or passive sensing by using hardware sensors (e.g., an acceleration sensor or camera sensor) (Sim 2019). Reducing effortful active sensing in place of more intelligent and unobtrusive approaches might be especially important against the background of achieving longevity. Context reasoning, which is also discussed under the label “digital biomarkers” in the medical literature, refers to using raw data for explaining and predicting contexts (e.g., psychological or physiological states) using data analytical methods (Perera et al. 2013; Sim 2019). For example, Sourial et al. (2016) use visual input for a hand therapy coach to predict the patient’s pain using image recognition of facial expressions when performing therapy exercises. Another example could be using GPS sensor data of the smartphone to predict the relapse risk of an obesity patient at places like the restaurant or supermarket. Future research could focus on similar unobtrusive approaches to capture and derive contextual data by making use of sensory capabilities (i.e., hardware of VC) in conjunction with AI (RQ16). However, battery and privacy aspects might play an important role in the acceptance of VCs when sensors of mobile devices are intensively used (see RQ12). Furthermore, up to this date, the entire potential and possible pitfalls of VCs have yet to be uncovered. Thus, identifying and systematically comparing similarities and differences of application areas constitutes an important area for future research (RQ17). For instance, what can be learned from a VC in the context of piano coaching for the design of a VC for fitness and vice versa.

**Table 2:** Theories that can inform the design of Virtual Coaches and exemplary research questions

Theory	Explanation	Exemplary research questions
Cognitive Load Theory **(**Sweller 1994, 2005**)**	Cognitive Load Theory states three additive factors that hamper learning: intrinsic (due to the natural complexity of the learning material), extraneous (due to inappropriate instructions), and germane cognitive load (effective efforts of the learner to understand the material). One main assumption of the theory is that the human working memory capacity is limited.	How should VCs take the users’ cognitive load into account? Under which conditions do VCs reduce cognitive load and improve coaching outcomes?
Social Cognitive Theory (Bandura 1986)	The Social Cognitive Theory assumes an interaction between personal, environmental, and behavioral factors that influence each other. Different theoretical constructs can be assigned to the three factors (e.g., self-efficacy and self-regulation to personal and observational learning to environmental factors).	How can peer coaching be integrated into VCs? Do peer coaching elements increase self-efficacy?
Transtheoretical Model **(**Prochaska and Velicer 1997**)**	The Transtheoretical Model is a stage model and assumes that people are passing different stages of change (SOC) during the behavior change process. In addition to the stages of change, there are also other theoretical constructs: processes of change (that promote the passage through the SOC’s), decisional balance (evaluating pros and cons of changing), self-efficacy, and temptation (opposite of self-efficacy).	How can the VC guide the user through the different SOCs? Does tailoring the coaching interventions to the SOC’s improve long-term behavior change?
Theory of Planned Behavior **(**Ajzen 1991**)**	Theory of Planned Behavior postulates that changes in behavior are influenced by one’s attitude towards the behavior, subjective norms, and perceived behavioral control. The behavioral intention mediates the three influencing variables.	How can the VC influence the coachee’s attitudes, subjective norms, and perceived behavioral control by using persuasive techniques?
Self Determination Theory **(**Ryan and Deci 2000**)**	Self Determination Theory attempts to explain that motivation for a certain behavior is influenced by the individual’s competence, autonomy, and relatedness.	How can the VC support competence, autonomy, and relatedness?
Capability, Opportunity, Motivation – Behavior Model (COM-B) **(**Michie et al. 2011**)**	The COM-B model assumes that the three factors (capability, opportunity, and motivation) mutually influence the behavior. The model is embedded into the core of the so-called “Behavior Change Wheel” (a higher-level framework), which points out several policy strategies and intervention functions (e.g., education, persuasion, incentives) for sustainable behavior change.	How can the intervention functions named by the behavior change wheel be effectively implemented in digital systems?
Computers are Social Actors paradigm/Social Response Theory **(**Nass and Moon 2000**)**	The CASA paradigm suggests that computers can influence cognition, affection, and behavior the same way as other people can. Humans apply social rules to computers and, thus, socially respond to certain anthropomorphic cues.	Will the VC’s anthropomorphic design lead to better coaching outcomes? What design elements will trigger certain social responses with respect to the coaching scenario?
Theory of Uncanny Valley (Mori et al. 2012**)**	The Theory of Uncanny Valley posits the idea that increasing the “humanness” by implementing an anthropomorphic design can increase the acceptance (affinity) at first, the acceptance can turn negative if the system appears “too human” but do not behave like a real human.	What are the limitations of an anthropomorphic design in VCs?

## Conclusion

In this catchword, we introduced the concept of VCs to IS research. We, therefore, synthesized the different understandings and overlapping of related concepts such as BCSSs or VAs (see Fig. 2). Further, we elaborated a framework that classifies the existing research into five building blocks (see Fig. 3). We investigated the related work for each block and suggested opportunities for the future research agenda. As shown in Table 3, several challenges beyond the technological complexity have to be mastered in the following years. Prospective research aspects can be found in all areas of the proposed research framework. In addition to specific questions related to the building blocks of VCs, there are overarching research questions for the IS community which address the societal and economic impact (e.g., on the transformation of established industries and digital life) (RQ18-20). Finally, demonstrating the evidence is crucial for the widespread adoption of VCs. A solid interdisciplinary discourse of technicians, IS researchers, psychologists and domain experts and a user-centered design is mandatory to develop effective solutions and maintain a long-term relationship. We hope that this catchword can jumpstart new collaborations and research projects by providing “food for thought” on how to approach the topic of VCs.

**Table 3:** A research agenda for virtual coaches

Research aspect	Exemplary research questions
A: Virtual Coach (VC)	RQ1: How can the VC be deployed on multiple devices (e.g., smartphone, smartwatch, VR/AR glasses) for combined application scenarios? RQ2: How can a “multi coach” approach be designed and implemented? RQ3: How can learning abilities of the system be implemented to personalize the coaching process? RQ4: Which XAI methods are particularly suitable for VCs to enhance user trust? RQ5: What are technical mechanisms to ensure the safety of the user? RQ6: How should generic virtual coaching solutions be designed?
B: User	RQ7: How can the constructs of psychological theories that explain learning and behavior change be effectively mapped to software systems (see Table 2)? RQ8: How can the VC support habit formation?
C: User—VC Interaction	RQ9: What are the long-term effects of certain social cues? RQ10: How can the VC be designed to effectively promote persuasiveness and user engagement? RQ11: How can a long-term human–VC relationship be established and maintained? RQ12: What are the consequences of an omnipresence of the coach? RQ13: How can ethical aspects be addressed when designing VCs?
D: Human Coach	RQ14: How should the interface for the human coach be designed? RQ15: How can human expert knowledge be efficiently integrated into the VC?
E: Context	RQ16: What are unobtrusive approaches to capture and predict the context? RQ17: What are application areas of VCs?
Overarching research questions	RQ18: Do VCs influence coaching-oriented business models (creating a coaching economy)? RQ19: Do VCs change established industries or other domains (e.g., healthcare)? RQ20: Will VCs help reduce inequalities (e.g., in education)?

## Footnotes

1 https://www.mobile-coach.eu/
